# Long-Term Suppressive cART Is Not Sufficient to Restore Intestinal Permeability and Gut Microbiota Compositional Changes

**DOI:** 10.3389/fimmu.2021.639291

**Published:** 2021-02-26

**Authors:** Giuseppe Ancona, Esther Merlini, Camilla Tincati, Alessandra Barassi, Andrea Calcagno, Matteo Augello, Valeria Bono, Francesca Bai, Elvira S. Cannizzo, Antonella d'Arminio Monforte, Giulia Marchetti

**Affiliations:** ^1^Clinic of Infectious Diseases, Department of Health Sciences, University of Milan, Azienda Socio Sanitaria Territoriale Santi Paolo e Carlo, Milan, Italy; ^2^Biochemistry Laboratory, Department of Health Sciences, University of Milan, Azienda Socio Sanitaria Territoriale Santi Paolo e Carlo, Milan, Italy; ^3^Unit of Infectious Diseases, Department of Medical Sciences, University of Turin, Turin, Italy

**Keywords:** dysbiosis, cART initiation, intestinal damage, microbial translocation, gut health

## Abstract

**Background:** We explored the long-term effects of cART on markers of gut damage, microbial translocation, and paired gut/blood microbiota composition, with a focus on the role exerted by different drug classes.

**Methods:** We enrolled 41 cART naïve HIV-infected subjects, undergoing blood and fecal sampling prior to cART (T0) and after 12 (T12) and 24 (T24) months of therapy. Fifteen HIV-uninfected individuals were enrolled as controls. We analyzed: (i) T-cell homeostasis (flow cytometry); (ii) microbial translocation (sCD14, EndoCab, 16S rDNA); (iii) intestinal permeability and damage markers (LAC/MAN, I-FABP, fecal calprotectin); (iv) plasma and fecal microbiota composition (alpha- and beta-diversity, relative abundance); (v) functional metagenome predictions (PICRUSt).

**Results:** Twelve and twenty four-month successful cART resulted in a rise in EndoCAb (*p* = 0.0001) and I-FABP (*p* = 0.039) vis-à-vis stable 16S rDNA, sCD14, calprotectin and LAC/MAN, along with reduced immune activation in the periphery. Furthermore, cART did not lead to substantial modifications of microbial composition in both plasma and feces and metabolic metagenome predictions. The stratification according to cART regimens revealed a feeble effect on microbiota composition in patients on NNRTI-based or INSTI-based regimens, but not PI-based regimens.

**Conclusions:** We hereby show that 24 months of viro-immunological effective cART, while containing peripheral hyperactivation, exerts only minor effects on the gastrointestinal tract. Persistent alteration of plasma markers indicative of gut structural and functional impairment seemingly parallels enduring fecal dysbiosis, irrespective of drug classes, with no effect on metabolic metagenome predictions.

## Background

HIV-infected individuals harbor a distinct gut microbiota (1–5) known to associate with immune activation (6–8), immune status (9–12), antiretroviral treatment (13, 14) and sexual orientation (15, 16). Despite the increasing recognition of the gut microbiome involvement in HIV pathogenesis (17–20), findings and interpretation of literature studies diverge quite significantly, due to differences in cohort size, sampling, lack of adjustment for confounding factors, such as sexual practice, age, and diet (13, 15).

One of the most consistent alterations described in untreated HIV infection is the dramatic subversion of the *Bacteroidetes* and *Proteobacteria* phyla, with, respectively, an unbalanced *Prevotella/Bacteroides* species ratio and enrichment in *Enterobacteriaceae* (19, 21–23). The increased abundance of gut-resident bacteria which are capable of directly driving inflammation in the host represents a reasonable mechanistic link between HIV-associated dysbiosis and high systemic immune activation in the natural history of disease (22).

Suppressive combination antiretroviral therapy (cART) appears to have a limited effect on the restoration of gut microbiota (1, 3, 12, 14, 23–27). Indeed, although gut microbial composition of cART-treated individuals is different from that of untreated subjects, the former also display a microbial community structure distinct from that reported in the HIV-uninfected population (3, 26, 28). These findings allow for the speculation that enduring gut dysbiosis may contribute to the pathogenesis of residual clinical disease in the course of cART. In addition, antiretroviral compounds *per se* could further promote dysbiosis (29) as well as impact on microbial translocation, inflammation/immune activation and the gut epithelial barrier damage (23, 24).

While focusing on a detailed definition of gut microbiota in HIV infection, literature studies have overlooked the possible role of blood microbiota, translocated from the gastrointestinal tract to the systemic circulation, in promoting inflammation and non-communicable diseases in HIV-infected subjects.

A previous report from our group demonstrated a polymicrobic flora in the blood of HIV-infected subjects with inadequate immune recovery on cART (30), raising the question of whether blood microbiota merely reflects the microbial composition in the gut or actively contributes to HIV pathogenesis. In keeping with this observation, recent studies have demonstrated a role of blood microbiota in the onset of diabetes and athero-thrombotic disease in the general population (31, 32), possibly confirming a role of blood dysbiosis in non-AIDS related co-morbidities (33).

In the attempt to shed light onto the mechanisms underlying gut dysbiosis, gastrointestinal damage, microbial translocation and systemic inflammation in the course of effective treatment, we conducted a longitudinal analysis of the microbial composition in paired blood and gut samples of HIV-infected subjects introducing cART and studied its association with microbial translocation, gastrointestinal damage and gut/systemic inflammation parameters.

## Materials and Methods

### Study Population

HIV-positive, adult, antiretroviral-naïve subjects were consecutively enrolled at the Clinic of Infectious Diseases, Department of Health Sciences, University of Milan—ASST Santi Paolo e Carlo, Milan, after providing written, informed consent in accordance with the Declaration of Helsinki. The Institutional Review Board at the ASST Santi Paolo e Carlo specifically approved the study. Individuals with either signs/symptoms of gastrointestinal diseases were excluded from the study. Study subjects underwent paired blood and fecal sampling prior to cART (T0) and after 12 (T12) and 24 (T24) months of therapy. HIV-uninfected individuals were enrolled as controls.

### T-Cell Immune Phenotypes

Lymphocyte surface phenotypes were evaluated by flow cytometry on fresh peripheral blood (FACSCanto II; BD Italy). We evaluated activation (CD45R0/CD38), naïve (CD45RA), and memory (CD45R0) subsets and IL-7 receptor (CD127) on CD4 and CD8 T-cells. Samples were stained with the following fluorochrome-labeled antibodies: L/D V500, CD4 PE-Cy7, CD8 PerCPCy5.5, CD38-FITC, CD45R0-PE, CD45RA-FITC, CD127-PE (BD Bioscience). The gating strategy used is presented in Supplementary Figure 1.

The following combinations were used: LD/CD8/CD38/CD45R0, LD/CD8/CD4/CD127, and LD/CD4/CD8/CD45RA/CD45R0. FACSDiva 6.1.3 software was used to analyze data.

### Microbial Translocation Markers

Plasma sCD14 and Endotoxin Core Antibodies (EndoCab) were measured by ELISA (R&D systems), in accordance with the manufacturer's instructions. Samples were diluted 1000X and 200X, respectively. The total amount of 16S rDNA present in the samples was measured by qPCR in triplicate and normalized using a plasmid-based standard scale (Vaiomer SAS, Labége, France).

### Urinary Lactulose-Mannitol Fractional Excretion Ratio (LAC/MAN) and Intestinal Fatty Acid Binding Protein (I-FABP)

Participants were asked to fast the night before and to collect morning urine before drinking a sugar probe solution containing 5 g lactulose and 2 g mannitol. Urine was collected for 5 h following administration of the double sugar solution and participants did not eat or drink (with the exception of water) until the end of the 5-h collection. The total volume of urine was recorded and a 30 mL aliquot of chlorhexidine-preserved (0.236 mg/mL of urine; Sigma Chemical, St Louis, MO, USA) was frozen and stored for High Performance Liquid Chromatography analysis of lactulose and mannitol (Dionex MA-1 ion exchange column with pulsed amperometric detection on a Dionex Ion Chromatograph 3000, Thermo Scientific, Sunnyvale, CA). The ratio of lactulose and mannitol excretion (LAC/MAN) was assessed. Normal values were considered <0.05 (34, 35). Intestinal Fatty Acid Binding Protein (I-FABP) was assessed by ELISA (Hycult Biotech), according to manufacturer's instruction.

### Fecal Calprotectin Levels

Calprotectin concentrations were measured by use of a commercial ELISA kit (Immundiagnostik, Bensheim, Germany), according to the manufacturer's instruction. Briefly, two 100-mg samples of feces from a single stool sample from each participant were assayed, and the mean of the two measurements was recorded.

### Gut Persistence Score

A gut persistence score was calculated for all drug regimens. Briefly, given the known bioavailability for every single cART molecule, the percentage of non-absorbed drug was ranked in quartiles and given a score between 1 (low persistence score, <25%) and 4 (high persistence score, >75%). A regimen gut persistence score was calculated as the sum of each ARV score.

### Metagenomic Sequencing of Blood and Fecal Samples

Fresh stool samples and plasma were collected from each subject, frozen immediately and stored until processing at −80°C. Total DNA was extracted as previously described (36). DNA from plasma was isolated and amplified in a strictly controlled environment at Vaiomer SAS (Labège, France) using a stringent contamination-aware approach as discussed previously (36–39), with no decontamination strategies.

However, plasma, which harbors only a small fraction of the blood bacterial DNA (39), could be impacted by technical contaminants, which suggests that plasma signatures should be taken with more caution than those obtained from feces. Potential contaminants usually impact all samples in a similar way and should not create artifactual differences in the statistical analyses.

Following DNA extraction, the V3–V4 hypervariable regions of the 16S rDNA were amplified and quantified by qPCR, sequenced with MiSeq technology, and clustered into operational taxonomic units (OTUs) before taxonomic assignment as described for fecal and plasma samples (36). PCR amplification was performed using 16S universal primers targeting the V3–V4 hypervariable region of the bacterial 16S ribosomal gene corresponding to 340F-781R sequence positions on the reference *E. coli* sequence.

The targeted metagenomic sequences from fecal and plasma microbiota were analyzed using the bioinformatics pipeline established by Vaiomer SAS from the FROGS guidelines. Briefly, after demultiplexing of the barcoded Illumina paired reads, single read sequences were cleaned and paired for each sample independently into longer fragments. Operational taxonomic units (OTU) were produced with via single-linkage clustering and taxonomic assignment was performed in order to determine community profiles. For parameters: the samples with <5,000 sequences after FROGS processing were not included in the statistics.

### Bioinformatics Analyses

Targeted metagenomic sequences from microbiota were analyzed using a bioinformatic pipeline based on FROGS (40), as described in (38). Briefly, the denoising was performed by removing amplicons missing the two PCR primer sequences (10% of mismatches were allowed), amplicons shorter than 350 bases or longer that 480 bases, amplicons with at least one ambiguous nucleotide (“N”), amplicons identified as chimera (with vsearch v1.9.5), and amplicons with a strong similarity (coverage and identity ≥ 80%) with the phiX genome (used as a control for Illumina sequencing runs). Clustering was produced in two passes of the swarm algorithm v2.1.6. The first pass was a clustering with an aggregation distance equal to 1. The second pass was a clustering with an aggregation distance equal to 3. As final denoising step, OTU with very low abundance (≤0.005%) were regarded as sequencing errors and thus discarded. Taxonomic assignment of amplicons into operational taxonomic units (OTUs) was produced by Blast+ v2.2.30+ with the RDP V11.4 database.

Samples with fewer than 5,000 sequences classified in OTU (3 plasma samples, and none of the fecal samples) were excluded from diversity and LEFSE analyses as their taxonomic profile was not determined with enough precision. However, no other cut off was used, and all other samples were kept in the analysis.

Reads obtained from the MiSeq sequencing system have been processed using Vaiomer SAS bioinformatics pipeline. The relative proportion taxa for each taxonomic level (phylum, class, order, family, genus, and species) for both fecal and plasma samples were analyzed statistically.

Alpha-diversity (α-diversity) represents the mean of species diversity per sample in each group/class. Diversity analysis is presented at OTUs level for richness parameters for species taxa according to (1) observed, (2) Chao1 and (3) PD (Phylogenetic Diversity) indexes; and diversity/evenness parameters for species taxa according to (3) Shannon, (4) Simpson, and (5) inverse Simpson indexes.

Principal Coordinate Analysis (PCoA) was performed for comparison of sample groups/class based on four methodologies for β-diversity: (1) Bray-Curtis (a quantitative measure of community dissimilarity), (2) Jaccard (a qualitative measure of community dissimilarity), (3) Unweighted-Unifrac (a qualitative measure of community dissimilarity that incorporates phylogenetic relationships between the features), and (4) Weighted-Unifrac (a quantitative measure of community dissimilarity that incorporates phylogenetic relationships between the features).

Finally, the output matrix containing the relative abundance of OTUs per sample was processed with the linear discriminant analysis effect size (LEfSe) algorithm (41) using an alpha cut-off of 0.05 for both the factorial Kruskal-Wallis test among classes and the pairwise Wilcoxon test between subclasses, and an effect size cut-off of 2.0 for the logarithmic LDA score for discriminative features, and the strategy for multi-class analysis set to “all-against-all.”

The functional metagenome has been predicted using PICRUSt v1.1.1 (42) as follow: the OTU representative sequences were used to pick OTUs against the GreenGenes reference tree (May 18, 2012 database) at 97% identity in order to convert our initial OTU abundance table into PICRUSt-ready OTU abundance table. The metagenome was predicted for each sample and the related pathways were retrieved from the Kyoto encyclopedia of genes and genomes (KEGG) pathways database and formatted for STAMP analysis.

### Statistical Analysis

Continuous variables were expressed as median and interquartile range (IQR), whereas categorical variables were expressed as absolute numbers and percentages. The different groups of patients and the different time points were compared using Chi-squared, Fisher's exact test for categorical variables. Mann-Whitney or Kruskal-Wallis for the comparison between HIV+ groups and HIV negative controls. Friedman paired test and Wilcoxon matched paired test for the comparison among HIV+ groups. Correlations among variables were tested by Spearman Rank correlation and presented through heatmaps created using the HEATPLOT module on Stata (v.14, StataCorp, USA). Data were analyzed with GraphPad 6.2 Prism (GraphPad Software Inc). Permanova analysis and Permdisp analysis for all Beta-diversity indexes were performed. *P* < 0.05 for both Pseudo F and *F-*values, respectively, for Permanova and Permdisp, were considered statistically significant. For Picrust analyses was used “STAMP analysis” using Welch's (uncorrected) 0.95 for *post-hoc* test, and Bonferroni correction.

## Results

### Study Population

We consecutively enrolled 41 HIV-infected, cART-naïve individuals (Table 1). At baseline, 25/41 (61%) started a NNRTI-based regimen, 9/41 (22%) a PI-based regimen and 7/41 (17%) an INSTI-based regimen (Table 1). Compared to HIV-negative healthy controls, HIV-infected patients were older (*p* = 0.0017), with a higher proportion of MSM (*p* = 0.039; Table 1).

**Table 1:** Epidemiological, Clinical, and HIV-related features of HIV-infected cohort.

	**HIV -infected (*N =* 41)**	**HIV negative (*N =* 15)**	***p-*value**
Age, years (IQR)^*^	42 (31.5–50.5)	29 (24–33)	**0.0017**
Sex, male (n) (%)°	37 (90)	15 (100)	0.56
Sexual behaviors MSM, n (%)°	28 (68.2)	5 (33.3)	**0.039**
BMI, n (IQR)^*^	22.66 (22.02–25.18)	22.71 (20.98–24.91)	0.86
Vegetarian/vegan diet, n subjects (%)°	3 (7.3)	0	0.55
Ongoing antibiotic prophylaxis, n (%)°	8 (19.5)	0	0.09
Hepatitis coinfection, n (%)°	2 (4.8)	0	1
First cART regimen, n (%)		n/a	n/a
NNRTI-based	25 (61)		
PI-based	9 (22)		
INSTI-based	7 (17)		

* *Data are median (IQR), statistical analysis Mann-Whitney test. °data are n (%), statistical analysis Fisher's Exact Test. IQR, Interquartile; cART, combination of antiretroviral therapy; NNRTI, non-nucleoside reverse transcriptase inhibitor; PI, protease inhibitor; INI, integrase inhibitor*.

Following 12 and 24 months of suppressive cART, we observed viro-immunological improvements (Supplementary Table 1), coupled with decreased T-cell activation and a redistribution of memory and naïve T-cell subsets (Supplementary Table 1). With regards to microbial translocation and gut barrier markers, cART introduction resulted in stable 16S rDNA and sCD14 levels, along with a rise in EndoCAb (*p* = 0.0001) and I-FABP plasma levels (*p* = 0.039; Supplementary Table 1). Interestingly, while fecal calprotectin was stable over time in the whole population (Supplementary Table 1), it significantly decreased in HIV-infected subjects with baseline calprotectin values above the range of normality (i.e., >50 μg/g) (168 mcg/g vs. 125 mcg/g vs. 60 mcg/g; *p* = 0.018). Likewise, despite a stable LAC/MAN ratio in the whole cohort (Supplementary Table 1), HIV-infected patients starting cART with a LAC/MAN ratio > 0.05 displayed a significant reduction at T12 (0.065 vs. 0.034; *p* = 0.031; Supplementary Table 1).

### Fecal Bacterial Composition Is Affected by Both HIV Infection and Sexual Behavior

We first assessed fecal alpha- and beta-diversity, as well as bacterial relative abundance between in HIV-infected cART-naive individuals and HIV-uninfected controls. The alpha-diversity richness indexes, but not the evenness indexes, were higher in HIV-infected subjects compared to the control group (observed: *p* = 0.029; Chao1: *p* = 0.011; PD: *p* = 0.08; Shannon *p* = 0.184; Simpson *p* = 0.303; Supplementary Figure 2A).

Following the fecal relative abundance analyses of the diverse taxonomic levels (phylum, class, order, family, genus, and species) HIV-positive individuals displayed higher *Actinomycetaceae* (*p* = 0.01), *Prevotellaceae* (*p* = 0.003), *Lactobacillaceae* (*p* = 0.003), *Peptococcaceae* (*p* < 0.0001), *Succinivibrionaceae* (*p* < 0.0001), *Fusobacteriaceae* (*p* = 0.01) and lower *Bacteroidaceae* (*p* < 0.0001), *Ruminococcaceae* (*p* = 0.066), and *Rikenellaceae* (*p* < 0.0001) (Supplementary Figure 2B) compared to controls. In particular, while we did not find major differences at the phylum level, we observed significant modification within the lower taxonomic levels, such as families and genera. These differences were confirmed by the linear discriminant analysis (LDA) effect size (LEfSe) with LDA score>2 as the cut-off, which also displayed the involvement of other taxa (Supplementary Figure 2C).

Given data on the effect of sexual behavior, particularly men who have sex with men (MSM), as a driving factor of large microbiome differences [15, 16], we decided to perform a sensitivity analysis within the HIV-infected group according to sexual behavior (i.e., 28 HIV+ MSM vs. 13 HIV+ MSW). Although not detecting differences in alpha-diversity, we confirm Bacteroidetes unbalance (Prevotellaceae-rich/Bacteroidaceae-poor) in MSM (Supplementary Figures 2E,F).

Of note however, when restricting the analysis in MSW from both HIV-infected and HIV-uninfected populations we show a microbial signature unique to HIV infection with significantly less Rikenellaceae (*p* = 0.012) and Ruminococcaceae (*p* = 0.012) (Supplementary Figures 2G,H) as well as higher Actinomycetaceae (*p* = 0.042), Lactobacillaceae (*p* = 0.012), and Succinivibrionaceae (*p* = 0.002) (highlighted in bold italics in Supplementary Figure 2D).

These unique microbial changes in HIV-infected subjects lead us to hypothesize diverse metagenomic functions. To answer this question, we used the bioinformatics tool PICRUSt (http://picrust.github.io/picrust), that revealed similar predicted functional metagenomic pathways in the two groups (Supplementary Figure 2H).

### Effect of 12 and 24 Months of Diverse cART Regimens on Fecal Bacteria Composition

We next asked whether diverse cART regimens might result in modifications of gut microbiota composition. To address this question, we first analyzed the impact of cART regimens overall, then we stratified our cohort according to the 3rd drug, i.e., NNRTI, PI, or INSTI.

**Figure 1:**
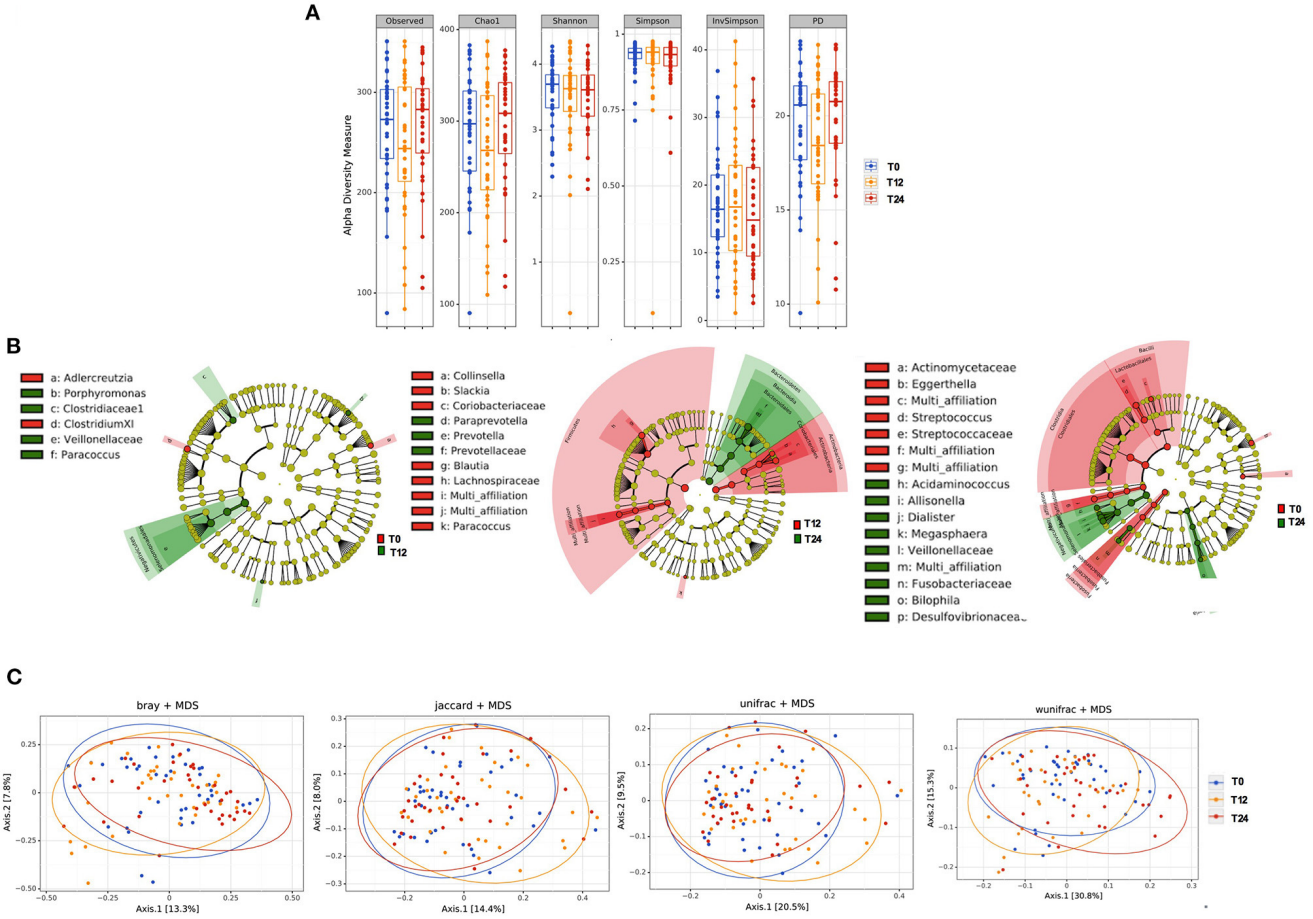
Fecal alpha and beta diversity analyses following 12 and 24 months of cART. **(A)** Fecal alpha diversity (α-diversity) is represented as the mean of species diversity per sample in each group/class (total OTUs) according to Observed, Chao1, PD (richness parameters), Shannon and Simpson (diversity/evenness parameters) during cART. We failed to find any changes following 12 (T12) and 24 months of cART (T24), data analyzed by Friedman test. **(B)** Linear discriminant analysis (LDA) effect size (LEfSe) with LDA score>2 log as the cut-off for all taxa at the following time-points: T0 in red and T12 in green (left); T12 in red and 24 months in green (center); T0 in red and T24 in green (right); all OTUs showed are significant with *p* < 0.05. **(C)** The principal coordinate analyses (PCoA) based on fecal beta-diversity results revealed no significant differences in the course of the study according to Bray-Curtis, Jaccard, unweighted-Unifrac; of note weighted Unifrac showed a significant separation between T12 and T24 (Permanova analysis pseudo *F* = 0.008), but not confirmed by Permdisp *F* = 0.92. *P-*values are not adjusted for multiple comparisons.

The initiation of antiretroviral therapy did not lead to substantial modifications of richness and evenness parameters, as shown in Figure 1A. Similarly, the beta-diversity LEfSe analyses showed a modest variation in gut bacterial composition following 12 and 24 months of cART (Figure 1B). In line with the above-mentioned results, the principal coordinate analyses based on beta-diversity results (Bray, Jaccard and Unifrac indexes) revealed minimal cluster changes of gut microbiota in the course of treated HIV infection, irrespective of cART duration (Figure 1C).

**Figure 2:**
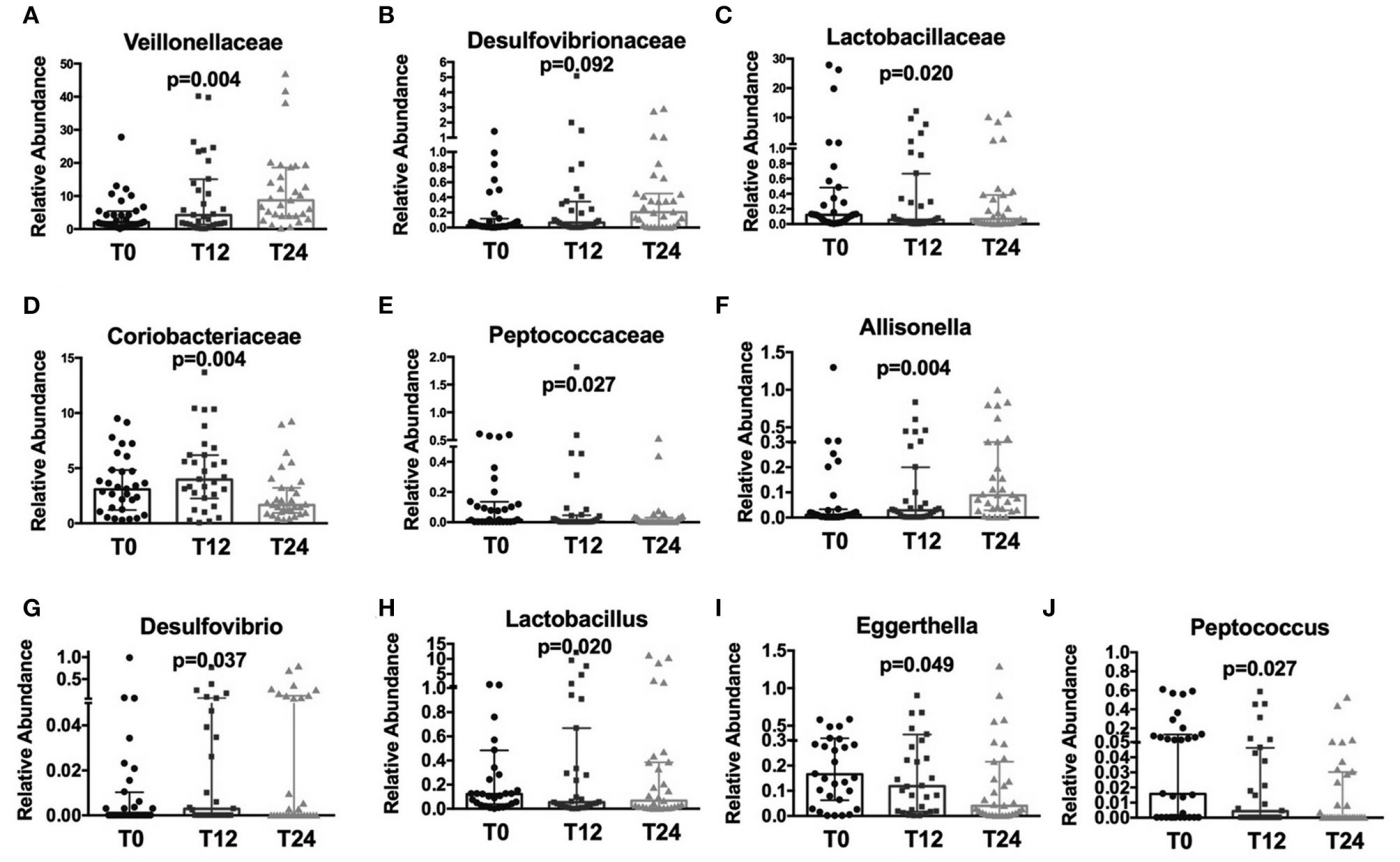
Fecal relative abundance of bacteria Family and Genus taxa following cART introduction. The presented data refer to taxa at family and genus levels in HIV -infected patients before (T0) and after cART (T12, T24). **(A)** Significant increase in *Veillonellaceae* family (*p* = 0.004). **(B)** Increasing trend in *Desulfovibrionaceae* (*p* = 0.92); **(C–E)** significant decrease, respectively, in *Lactobacillaceae, Coriobacteriaceae*, and *Peptococcaceae* families (*p* = 0.020, *p* = 0.004, *p* = 0.027, respectively). **(F,G)** Significant increase in Allisonella and Desulfovibrio genera (*p* = 0.004, *p* = 0.037, respectively). **(H–J)** Significant decrease, respectively, in Lactobacillus, Eggerthella, and Peptococcus genera (*p* = 0.020, *p* = 0.049, *p* = 0.027, respectively). Data analyzed by Anova, *p-*values are not adjusted for multiple comparisons.

Following cART, the relative abundance analysis revealed a significant increase in *Veillonellaceae* (*p* = 0.004; Figure 2A) and a non-significant trend toward higher *Desulfovibrionaceae* (*p* = 0.092; Figure 2B), coupled with a parallel decrease in *Lactobacillaceae* (*p* = 0.020), *Coriobacteriaceae* (*p* = 0.004), and *Peptococcaceae* (*p* = 0.027); Figures 2C–E). Furthermore, at genus level we observed an increase in *Allisonella* (*p* = 0.004), and *Desulfovibrio* (*p* = 0.037); Figures 2F,G), with a significant decrease in *Lactobacillus* (*p* = 0.020), *Eggerthella* (*p* = 0.049), and *Peptococcus* (*p* = 0.027) (Figures 2H–J).

Because we initially demonstrated an influence of sexual behavior on fecal microbiome, in a sensitivity analysis we decided to investigate whether sexual orientation still accounts for fecal composition following 12 and 24- month cART. Most interestingly, MSM and MSW confirmed higher *Prevotellaceae* along with significant differences in the distribution of *Bifidobacteriaceae, Bacteroideaceae, Succinivibrionaceae* and *Desulfovibrionaceae* (Supplementary Figures 3A–H).

**Figure 3:**
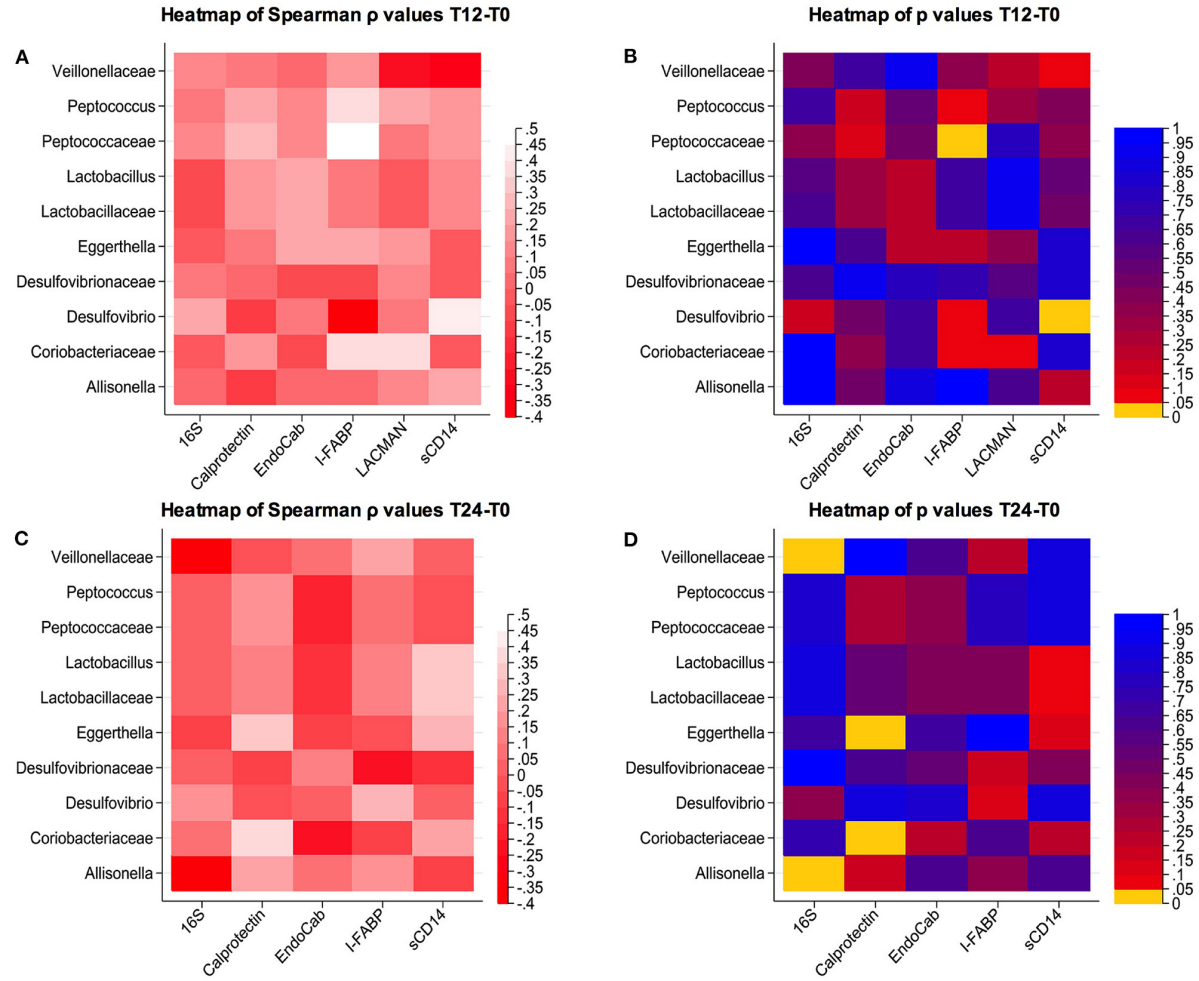
Correlations between changes in microbial composition and soluble biomarkers. **(A,B)** Spearman correlation and *p-*values at T12-T0; **(C,D)** Spearman correlation and *p-*values at T24-T0.

We next explored whether these cART-mediated microbiota shifts might be associated with markers of gut damage, microbial translocation, confirming possible associations between bacterial composition and their changes over time and gut damage/microbial translocation (Figure 3).

### Changes in Gut Microbiota Composition According to the cART Class

**Figure 4:**
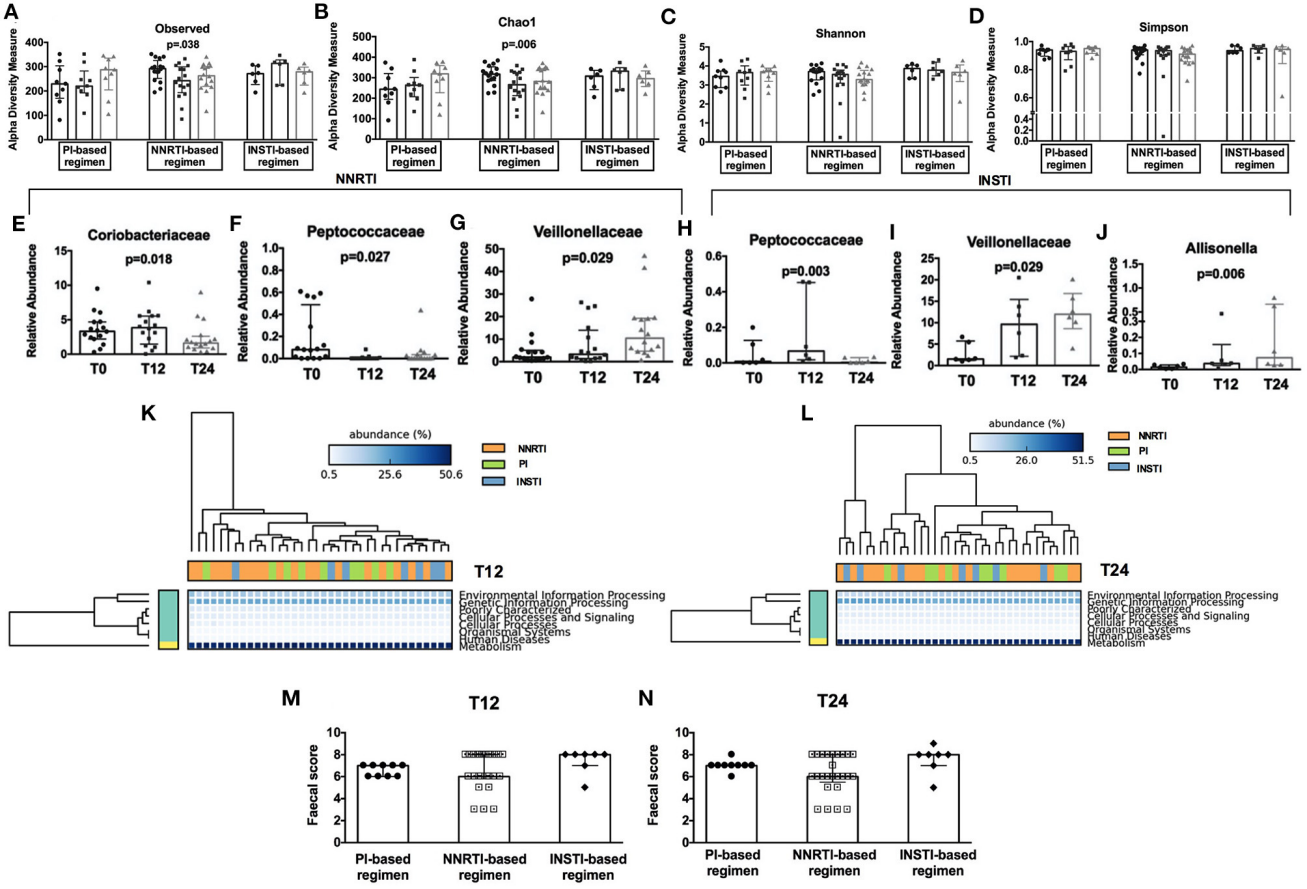
Fecal alpha diversity indexes, relative abundance analyses, metagenomic prediction, and gut persistence score according to cART class. Comparison of alpha diversity measures in HIV-infected patients during cART (3 bars, from left to right: T0, T12, and T24) subdivided according to different cART regimens presented according to Observed **(A)**, Chao1 **(B)**, Shannon **(C)**, Simpson **(D)** at OTUs level. **(E–J)** Comparison of fecal relative abundance taxa according to cART regimens analyzed Friedman test, *p-*values not adjusted for multiple comparisons. **(K,L)** Predicted metagenomic functions according to class cART. Data analyzed by Welch's (uncorrected) 0.95 for *post-hoc* test, and Bonferroni correction. **(M,N)** No differences were found in terms of fecal Gut Persistence Score according to class cART in the course of the study analyzed by Friedman test, *p-*values are not adjusted for multiple comparisons.

Interestingly, when we stratified patients according to cART regimens, we found that only NNRTI-based therapy significantly reduced richness (observed: *p* = 0.038; Chao1: *p* = 0.006; Figures 4A,B), but not evenness indexes (Figures 4C,D) over time. Furthermore, the relative abundance analyses showed a different profile at both family and genus levels, with NNRTI-based regimens significantly reducing the families of *Coriobacteriaceae, Peptococcaceae* and increasing the *Veillonellaceae* family (Figures 4E–G). At the opposite, INSTI-based regimens resulted in decreased *Peptococcaceae* and increased *Veillonellaceae* families, as well as in higher *Allisonella* genus (Figures 4H–J). Interestingly, the changes in gut dysbiosis according to diverse cART regimens, was accompanied by stable metagenomics predictions (Figures 4K,L).

Having shown fecal microbiome changes specific to drug class, we next aimed to test whether this effect could be associated to differences in gut drug persistence across drug classes by calculating a “gut persistence score” (GPS), based on the drug bioavailability and the proportion of unchanged drug in feces. We found that the gut persistence score was similar between PI-, NNRTI- and INSTI-based regimens over time (Figures 4M,N), overall suggesting that, despite similar drug concentration in the gut, cART classes are associated with different microbiome signatures, not translating however in different predictive metagenomic functions.

### Bacteria Alpha- and Beta-Diversity and Relative Abundance Analyses in Plasma

Given the presence of microbial bioproducts within the blood of HIV-infected individuals despite cART introduction (30, 43, 44), we also explored the composition of the translocating microbiota in plasma samples of our study cohort.

**Figure 5:**
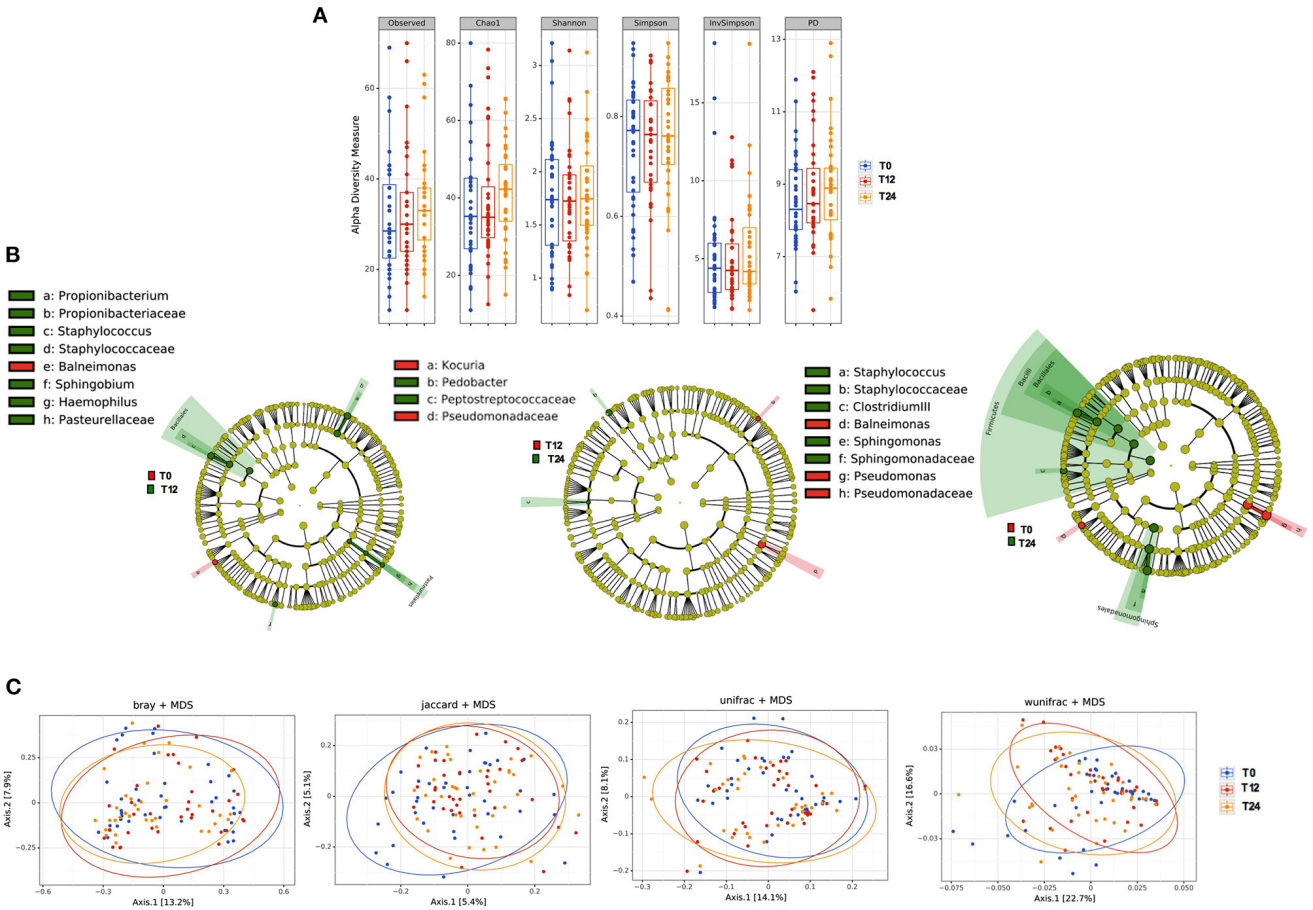
Plasma microbiota composition: alpha and beta diversity analyses. **(A)** Plasma alpha diversity (α-diversity) represented as the mean of species diversity (total OTUs) per sample in each group/class according to Observed, Chao1, PD (richness parameters), Shannon, Simpson, and invSimpson (diversity/evenness parameters) before (T0) and after 12 (T12) and 24 (T24) months of cART. No significant differences following cART initiation were found (Friedman test). **(B)** Linear discriminant analysis (LDA) effect size (LEfSe) with LDA score>2 log as the cut-off for all taxa at the different time-points in study between baseline (T0) in red and 12 months (T12) in green of cART (left), T12 in red and 24 months (T24) in green of cART (center), T0 in red and T24 in green (right), all OTUs showed are significant with *p* < 0.05. **(C)** The principal coordinate analyses (PCoA) based on plasma beta-diversity results revealed no significant differences in the course of the study according to all parameters: BrayCurtis Permanova Pseudo *F* = 0.49, Perdsp. *F* = 0.32; Jaccard, pseudo *F* = 0.94, *F* = 0.14; unweighted Unifrac Pseudo *F* = 0.99, *F* = 0.85; weighted Unifrac 0.09, *F* = 0.50. *P-*values are not adjusted for multiple comparisons.

No differences were observed in richness parameters (observed, Chao1 and PD) and diversity/evenness parameters (Shannon and Simpson) in terms of bacteria composition over time (Figure 5A). In line with what observed in the feces, cART introduction modified the bacteria composition only partially, with slight variations of *Bacilli* genus (*p* = 0.03), *Sphingomonadales* order (*p* = 0.01) and *Sphingomonadaceae* family (*p* = 0.04) (Figure 5B). Similarly, beta- diversity analysis revealed no differences in plasma bacteria composition following the principal coordinate analysis during cART (Figure 5C).

Paired plasma and fecal subjects showed a completely different bacterial composition at phylum level, irrespective of HIV infection or cART introduction (Supplementary Figure 4).

## Discussion

It is now widely accepted that, aside from the direct effect on gastrointestinal mucosal immunity (45–48), HIV infection is characterized by gut microbiota compositional and functional changes, not fully reverted by cART (1, 3, 5, 12, 13, 18, 49–53). However, the causal relationship between altered intestinal microbiota composition, gut damage, and cART remains an open question that needs to be answered in order to improve microbiome-targeted therapies.

We hereby describe that 24 months of viro-immunological effective cART significantly reduced immune activation and corrected T-cell immune phenotype imbalances in the periphery, yet appeared to have a minor effect within the gut. Literature reports have demonstrated greater IFAB-P levels in HIV infection (54). Although IFAB-P measurements in HIV-uninfected subjects were not available in the present study, our results of a significant rise in plasma I-FABP during 24-month cART possibly suggest enduring enterocyte damage despite long-term cART. These findings complement previous data by Chevalier et al. (54) on the rise of I-FABP in acutely treated HIV-infected patients, implying the inefficiency of cART in preventing/correcting gut mucosal damage either in acute and in chronic infection. In keeping with this observation, the findings of a rise in both I-FABP and EndoCAb in the face of stable 16S rDNA might altogether suggest ongoing intestinal epithelium compromise, in turn conditioning the release in the systemic circulation of bacteria products that might however be cleared by naturally occurring EndoCAbs as previously suggested (55). Under this perspective, the finding of stable plasma sCD14 over 24 months is therefore not unexpected, given that sCD14 has been proven to interact with translocating bacteria products, in turn stimulating antigen-presenting cells via TLR signaling (56).

Although specific clinical indexes of gut permeability (i.e., LAC/MAN and fecal calprotectin) slightly and selectively improved in patients starting cART with advanced gut damage, our data overall point to the unproductive attempt of cART to repair the mucosal barrier.

The persistence of gut damage during long-term cART appears to mirror the limited effects of treatment on fecal microbiota composition, with modest increases in *Negativicutes, Selenomonadales, Veillonellaceae* and decreases in *Lactobacillaceae, Peptococcaceae, Coriobacteriaceae*. While some of these modifications might be helpful in restoring the balance between microbiota and immune system (57, 58), other changes, such as the emergence of *Allisonella* or *Desulfovibrio* genera, coupled with the possible positive association between the *Veillonellaceae* family and I-FABP levels, might indicate a perpetuation of gut damage. Further studies to finely investigate the associations between microbiome compositional signatures in both the stool and in gut tissue and markers of gut damage will be needed to better comprehend the possible interactions between gut microbiome and damage, in turn possibly affecting disease progression (59, 60).

Indeed, in line with previous literature reports (3, 12, 26, 28, 50), our cohort of chronically HIV-infected individuals maintained gut dysbiosis, featuring higher fecal α-diversity, a distinct cluster separation according to PCoA analysis and a *Prevotellaceae*-rich/*Bacteroidaceae* poor profile compared to uninfected individuals as well as a Ruminococcaceae/Rikenellaceae poor profile in MSW alone. Our finding is coherent with other authors, suggesting a complex scenario where the gut microbiota is altered by both HIV and other confounding factors (61).

Given that the enrichment or impoverishment of some key microbial species is linked to disease progression—i.e., the depletion of butyrate-producing bacteria has been associated with increased microbial translocation and immune activation (62, 63)–, our findings support the role of specific bacterial populations in the pathogenesis of non-communicable disorders in the context of treated HIV infection. Additionally, our finding of higher fecal α-diversity in HIV-infected patients seems to be in contrast with previous studies (12) and might be attributed in part to differences in sexual practices. Indeed, MSM were found to have greater fecal microbial differences than not-MSM, irrespective of HIV infection (15). In our cohort, however, the sensitive analysis on not-MSM confirmed the differences between HIV-infected and HIV- individuals, suggesting that factors other than sexual practice might have influenced the microbiota composition.

Antiretroviral drugs may contribute to the development of non-infectious comorbidities because of their widely described adverse effects on various organs and systems; further, cART may also determine compositional shifts in the gut microbiome and possibly fuel disease progression. In our study, aside from a small NNRTI-mediated decrease in α-diversity richness, we did not highlight marked changes according to PI- or INSTI-based combinations. Although in contrast with literature observations showing a possible role of cART-associated modifications in microbial composition (15, 23), the different cART regimens appear to have a similar impact on the composition of the microbiota.

We should also acknowledge that, given the low CD4 count at cART initiation, 19% of our cohort was on antibiotic prophylaxis, that was promptly interrupted when patients reached a good immune recovery level (generally within the first year). Thus, we could not exclude that part of the small modifications observed in gut microbiota composition might be mediated by antibiotic suspension. Besides, some cART regimens, particularly protease inhibitor-based combinations, could induce non-infectious diarrhea (64, 65), so it is also possible that the changes in microbiota might reflect these side-effects.

We next focused our research on the characterization of blood microbiota composition, given the role of translocated microbial bioproducts in disease progression (30, 44). Our analyses revealed a different composition of translocated microbial products between plasma and fecal samples, in terms of both alpha and beta-diversity, suggesting, on the one hand, a selective passage of microbes through the gut barrier, and on the other, immune control over potentially pathogenic microorganisms. Interestingly, we describe predominance of the *Proteobacteria* phylum in plasma in contrast to its low intestinal abundance both before and after the introduction of cART. Our findings add to previous research describing the tempo and the signature of blood microbiota in both treated and untreated SIV and HIV-infected patients, and their possible influence on inflammation and immune homeostasis (21, 66, 67). Strikingly, a similar selective *Proteobacteria* overgrowth has been previously proven in the blood of experimentally SIV-infected asian macaques upon antiretroviral treatment, also associated with their increased metabolic activity within the gut and immune activation, altogether confirming the propensity to preferentially translocate in the systemic circulation, in turn conditioning the immune profile (21). Given that *Proteobacteria* in the blood have been associated with the onset of cardiovascular events (32), a detailed definition of the possible associations between the bacteria belonging to the *Proteobacteria* phylum and clinical outcomes in HIV-treated patients merits an in-depth investigation, to circumstantiate the role of dysbiosis in the development of residual disease during cART as recently suggested (68).

Several limitations in our study should be acknowledged: (i) our inability to study bacterial composition, gut damage and immunity at gut mucosal site, that might have shed light on the causal relationship between microbiota and immune system over the course of cART, (ii) the lack of an extensive analysis of microbial function, which would certainly widen the understanding of the possible interactions between gut microbial community and damage, as well as peripheral immune homeostasis, (iii) the lack of a standardized questionnaire on food habits, and (iv) the use of a non-validated drug penetration score into the feces, given the well-known distinctive ability of tissue penetrations exerts by the various drugs (69, 70). A further bias in our study might be represented by the younger age in control group. It is well-known that aging affects gut microbiota composition (71), however during adulthood (25–50 years) the composition tends to be stable (72, 73), thus we could assume that despite the statistical difference in age, the two study groups are homogeneous. Finally, we must acknowledge that specific plasma decontamination strategies were not applied in this data set. Given the possibility of plasma environmental contamination (39), although potential contaminants usually impact all samples in a similar way, they cannot be fully ruled out, and therefore microbial signatures in blood should be taken with more caution than those described in fecal samples.

In conclusion, despite the viro-immunological benefits, long-term effective cART, irrespective of drug classes, resulted in persistent gut damage that associates with gut dysbiosis. Besides, the finding of a compositional shift in fecal vs. plasma microbiota, with the enrichment of *Proteobacteria* in peripheral blood, opens new perspective on the clinical implication of circulating bacteria and HIV-associated non-communicable co-morbidities.

To our knowledge, this is the first study assessing the impact of 24 months of cART on both fecal and plasma microbiota composition. Whether the persistence of dysbiosis fuels intestinal damage and the consequent microbial translocation, or whether the HIV-mediated pro-inflammatory environment linked to gastrointestinal damage and microbial translocation promote dysbiosis remains to be elucidated.

## Data Availability Statement

The datasets presented in this study can be found in online repositories. The names of the repository/repositories and accession number(s) can be found at: ENA database under the project accession PRJEB41869.

## Ethics Statement

The studies involving human participants were reviewed and approved by Ethics committee of ASST San Paolo e Carlo. The patients/participants provided their written informed consent to participate in this study.

## Author Contributions

GA and EM designed and performed experiments, analyzed and interpreted the data, and wrote the manuscript. AB was responsible for HPLC data. CT, FB, and AC helped in analyzing and interpreting the data. EC and VB participated to laboratory experiments. MA helped with patients recruitment and manuscript preparation. Ad'A contributed to data interpretation. GM conceived and designed the study and conducted the data analyses. All authors contributed to the editing of the manuscript. GM supervised the project.

## Conflict of Interest

The authors declare that the research was conducted in the absence of any commercial or financial relationships that could be construed as a potential conflict of interest.

## Supplementary Material

Supplementary Figure 1 Gating strategy for the identification of CD4^+^ and CD8^+^ T-cell surface phenotypes. Lymphocytes were gated from forward (FSC) and side scatters (SSC), doublets were removed, live cells were selected and segregated for CD4^+^ T-cells or CD8 T-cells. Within CD4^+^ (or CD8^+^) subsets, CD127, CD45RA, CD45R0, and/or CD38 gates were set up based on positive vs. negative peak. Depicted are representative plots from representative subject PBMCs.

Supplementary Figure 2 **(A)** Fecal alpha diversity (α-diversity) represented as the mean of species diversity per sample in each group/class (total OTUs) according to Observed, Chao1 (richness parameters), Shannon and Simpson (diversity/evenness parameters) at baseline. We found a significant increase in HIV-infected patients compared to controls according to richness parameters (Observed *p* = 0.029; Chao1: *p* = 0.011, Mann-Whitney test); no differences according to diversity/evenness parameters (Shannon *p* = 0.184; Simpson *p* = 0.3033) were observed. **(B)** Fecal relative abundance at each taxonomic level (phylum, class, order, family, genus) between HIV-infected individuals at T0 and HIV-uninfected controls. ↑ indicates increase with significant *p* < 0.05, ↓ indicates decrease with significant values < 0.05 with exception for taxa Bifidobacteriales, Carnobacteriaceae, Acidaminococcus, Bifidobacterium, Collinsella, and Granulicatella that show trends with *p-*values between 0.05 and 0.07. Data analyzed by Mann-Whitney test. **(C)** Linear discriminant analysis (LDA) effect size (LEfSe) with LDA score>2 log as the cut-off at baseline. Significant results (*p* < 0.05) for all taxa are shown: higher abundance in HIV-infected subjects and HIV-uninfected controls represented in red and green, respectively. In Bold Italics are highlighted the families that remained altered in the sensitivity analysis according to sexual preferences (HIV-infected vs. HIV-uninfected men who have sex with women_MSW). **(D–G)** Sensitivity analysis of fecal microbial abundance according to sexual behavior and HIV-serostatus. HIV-infected men with different sexual behavior showed different microbial composition prior to cART (T0), with higher Prevotellaceae and lower Bacteriodaceae in MSM **(D,E)**. HIV-infected, cART-naive (T0) MSW displayed significantly lower Rikenellaceae and Ruminococcaceae compared to their sero-negative counterparts **(F,G)**. Data analyzed by Mann-Whitney test, *p* < 0.05. *P-*values are not adjusted for multiple comparisons. **(H)** Computed metagenomic functions between HIV-infected subjects prior to cART and HIV-uninfected subjects. Despite different microbial composition, similar functions were found in HIV-infected subjects prior to cART introduction and HIV-uninfected controls. Data anylzed by Welch's (uncorrected) 0.95 for *post-hoc* test, and Bonferroni correction.

Supplementary Figure 3 Sensitivity analysis of fecal microbial abundance according to sexual behavior following 12 and 24 months of cART. HIV-infected men with different sexual behavior showed different microbial composition at a family taxa level during cART both at T12 and T24. MSM showed lower Bifidobacteriaceae compared to MSW at T12 **(A)** and T24 **(E)**, and higher Peptococcaceae, Prevotellaceae, and Succinivibrionaceae both at T12 **(B–D)** and at T24 **(F–H)**. Data analyzed Mann-Whitney test, *p* < 0.05. *P-*values are not adjusted for multiple comparisons.

Supplementary Figure 4 Fecal and Plasma Phyla Distribution. The figure shows the relative abundance ratio between fecal (left bars) and plasma (right bars) samples among the most 4 representative phyla: Actinobacteria **(A)**, Proteobacteria **(B)**, Firmicutes **(C)**, and Bacteroidetes **(D)** for each group: HIV negative controls, HIV+ T0 (baseline), HIV+ T12 (after 12 months the introduction of cART) and HIV+ T24 (after 24 months the introduction of cART). **(A,B)** The relative abundance of Actinobacteria and Proteobacteria phyla was higher in plasma (right bars) than in feces (left bars) in all the study groups. **(C,D)** The relative abundance of Firmicutes and Bacteroidetes phyla was lower in plasma (right bars), as compared to feces (left bars), in both healthy controls and HIV-infected patients prior and after cART introduction. *P-*values are not adjusted for multiple comparisons.

Supplementary Table 1 Viro-immunological parameters, T-cell phenotypes, microbial translocation, and gut barrier markers in 41 HIV-infected patients starting a first cART regimen.

Supplementary Table 2 Viro-immunological parameters, T-cell phenotypes, microbial translocation/gut barrier markers in HIV-infected subjects treated with different classes of antiretrovirals.
