# Supplementary figures and images for: Long-Term Suppressive cART Is Not Sufficient to Restore Intestinal Permeability and Gut Microbiota Compositional Changes
Source: Front Immunol. 2021 Feb 26;12:639291. doi: 10.3389/fimmu.2021.639291 (PMC7952451; doi:10.3389/fimmu.2021.639291)

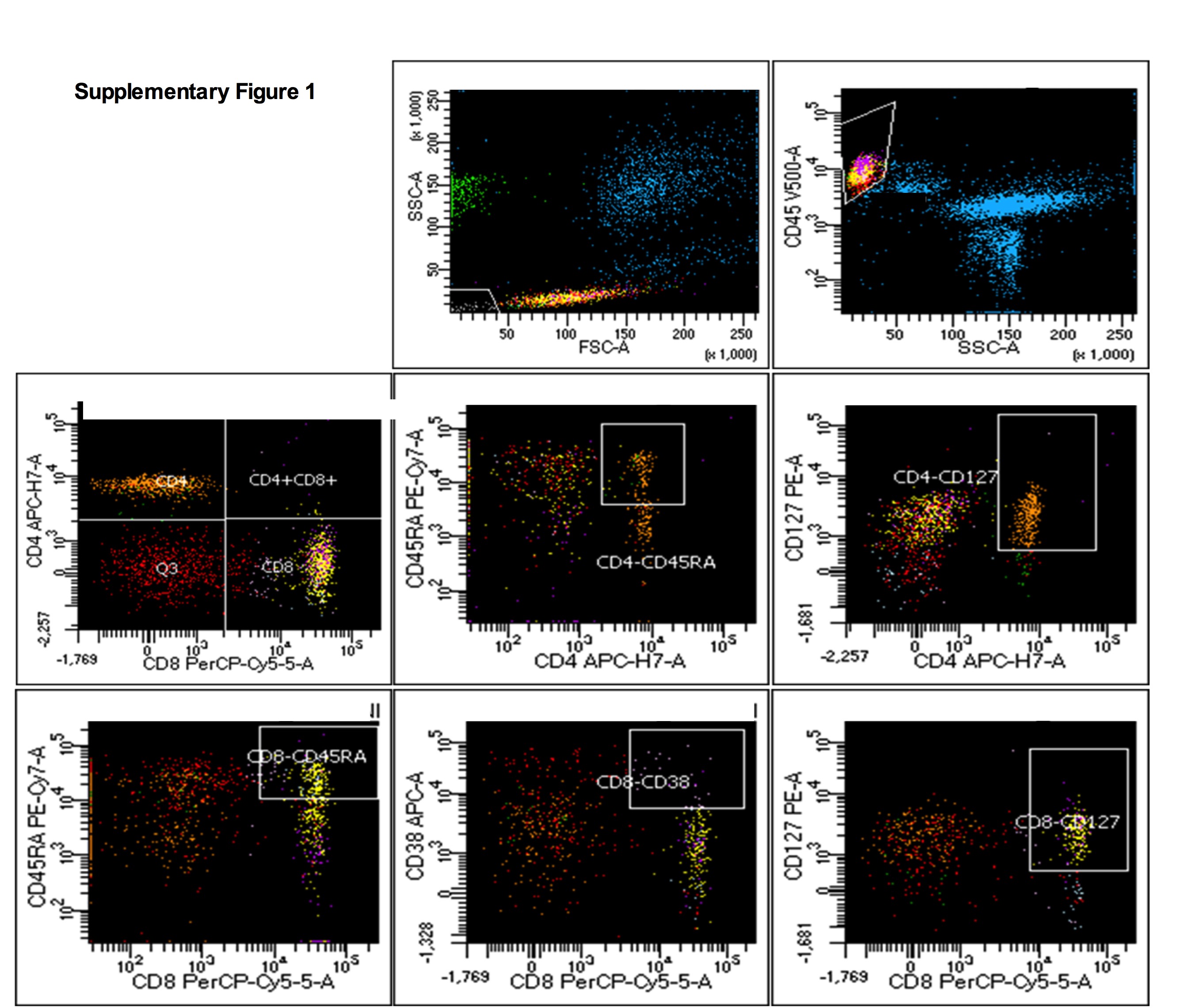

Supplement: Supplementary Figure 1 — Gating strategy for the identification of CD4+ and CD8+ T-cell surface phenotypes. Lymphocytes were gated from forward (FSC) and side scatters (SSC), doublets were removed, live cells were selected and segregated for CD4+ T-cells or CD8 T-cells. Within CD4+ (or CD8+) subsets, CD127, CD45RA, CD45R0, and/or CD38 gates were set up based on positive vs. negative peak. Depicted are representative plots from representative subject PBMCs. [file Image_1.JPEG]

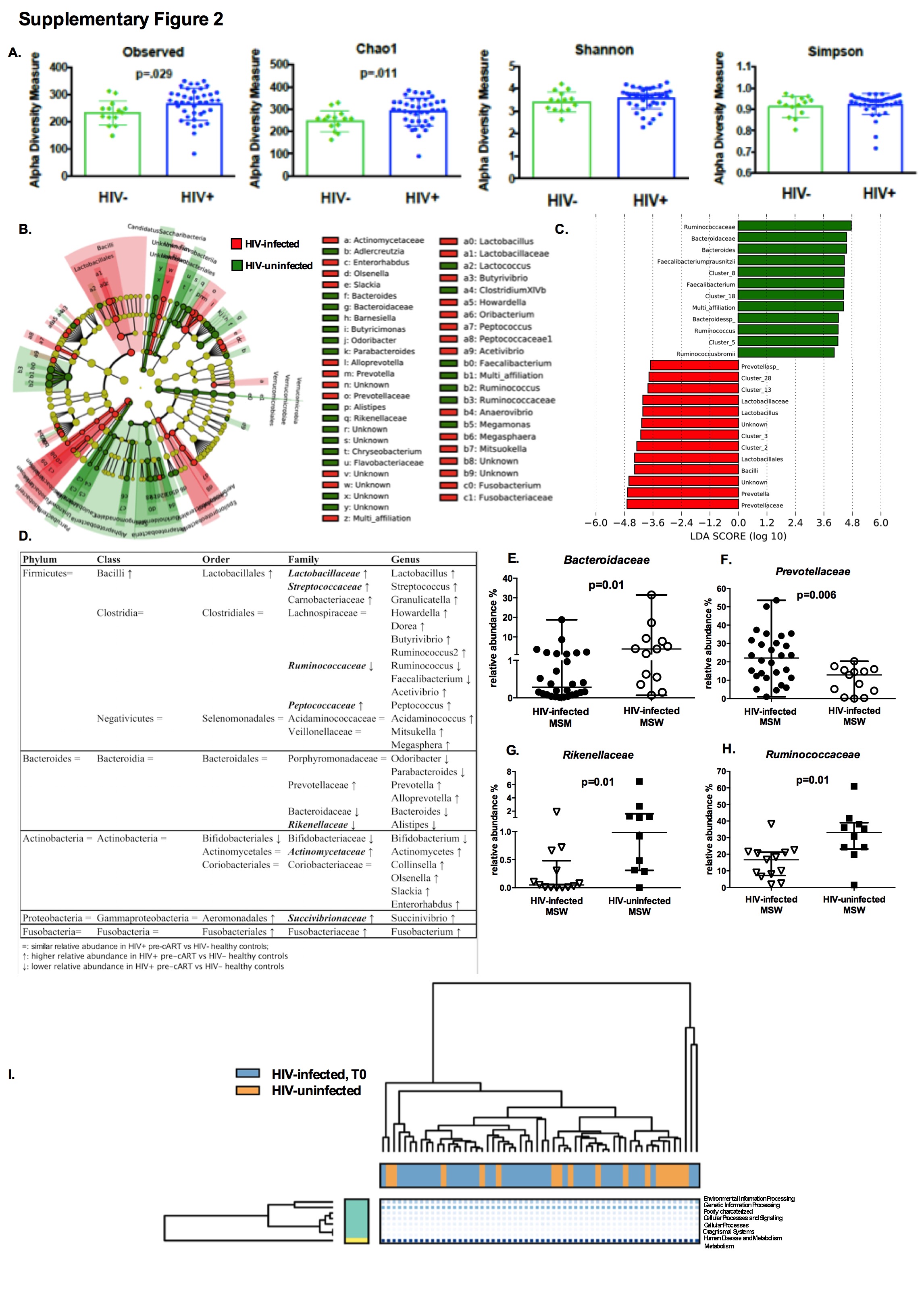

Supplement: Supplementary Figure 2 — (A) Fecal alpha diversity (α-diversity) represented as the mean of species diversity per sample in each group/class (total OTUs) according to Observed, Chao1 (richness parameters), Shannon and Simpson (diversity/evenness parameters) at baseline. We found a significant increase in HIV-infected patients compared to controls according to richness parameters (Observed p = 0.029; Chao1: p = 0.011, Mann-Whitney test); no differences according to diversity/evenness parameters (Shannon p = 0.184; Simpson p = 0.3033) were observed. (B) Fecal relative abundance at each taxonomic level (phylum, class, order, family, genus) between HIV-infected individuals at T0 and HIV-uninfected controls. ↑ indicates increase with significant p < 0.05, ↓ indicates decrease with significant values < 0.05 with exception for taxa Bifidobacteriales, Carnobacteriaceae, Acidaminococcus, Bifidobacterium, Collinsella, and Granulicatella that show trends with p-values between 0.05 and 0.07. Data analyzed by Mann-Whitney test. (C) Linear discriminant analysis (LDA) effect size (LEfSe) with LDA score>2 log as the cut-off at baseline. Significant results (p < 0.05) for all taxa are shown: higher abundance in HIV-infected subjects and HIV-uninfected controls represented in red and green, respectively. In Bold Italics are highlighted the families that remained altered in the sensitivity analysis according to sexual preferences (HIV-infected vs. HIV-uninfected men who have sex with women_MSW). (D–G) Sensitivity analysis of fecal microbial abundance according to sexual behavior and HIV-serostatus. HIV-infected men with different sexual behavior showed different microbial composition prior to cART (T0), with higher Prevotellaceae and lower Bacteriodaceae in MSM (D,E). HIV-infected, cART-naive (T0) MSW displayed significantly lower Rikenellaceae and Ruminococcaceae compared to their sero-negative counterparts (F,G). Data analyzed by Mann-Whitney test, p < 0.05. P-values are not adjusted for multiple comparison [file Image_2.JPEG]

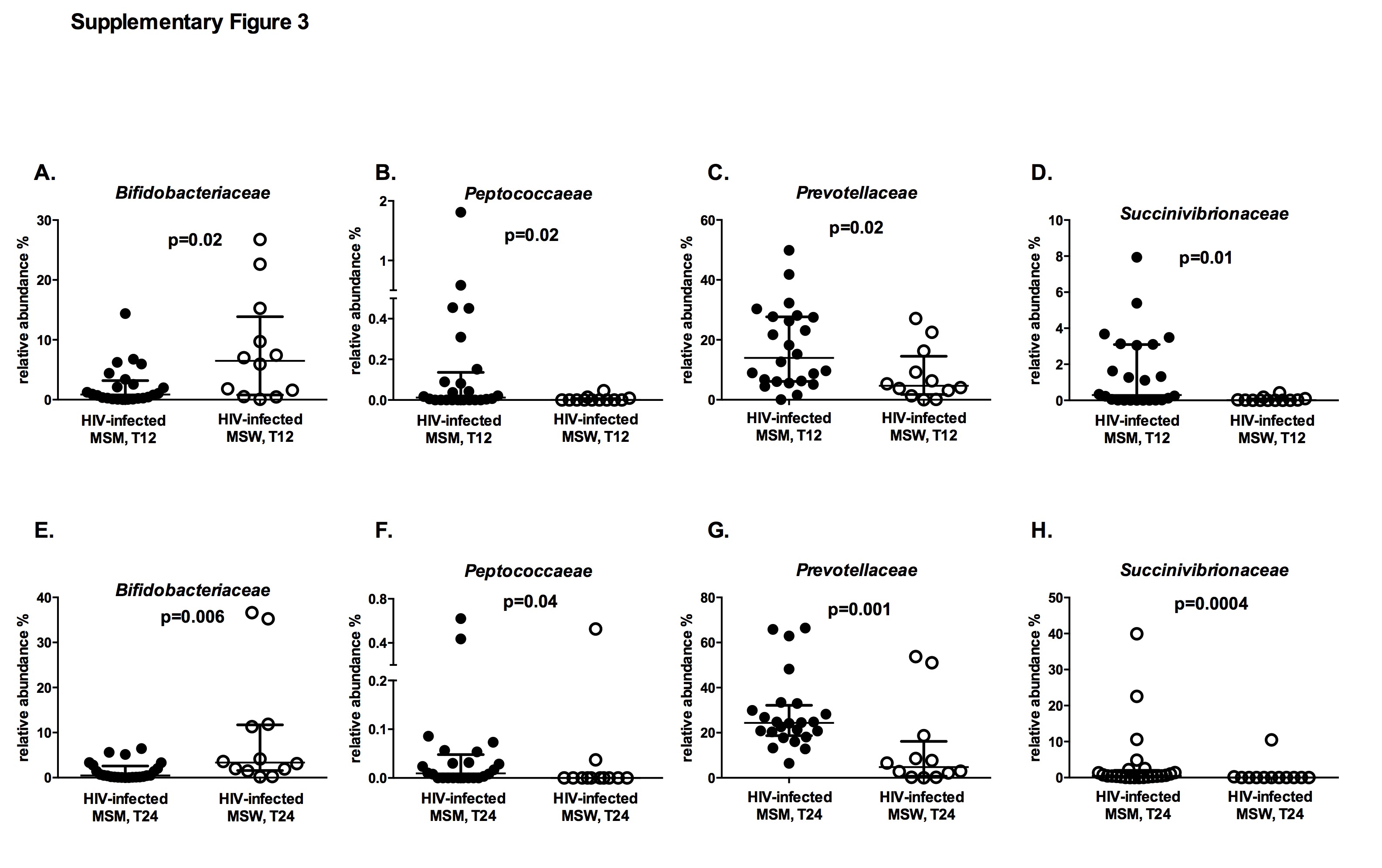

Supplement: Supplementary Figure 3 — Sensitivity analysis of fecal microbial abundance according to sexual behavior following 12 and 24 months of cART. HIV-infected men with different sexual behavior showed different microbial composition at a family taxa level during cART both at T12 and T24. MSM showed lower Bifidobacteriaceae compared to MSW at T12 (A) and T24 (E), and higher Peptococcaceae, Prevotellaceae, and Succinivibrionaceae both at T12 (B–D) and at T24 (F–H). Data analyzed Mann-Whitney test, p < 0.05. P-values are not adjusted for multiple comparisons. [file Image_3.JPEG]

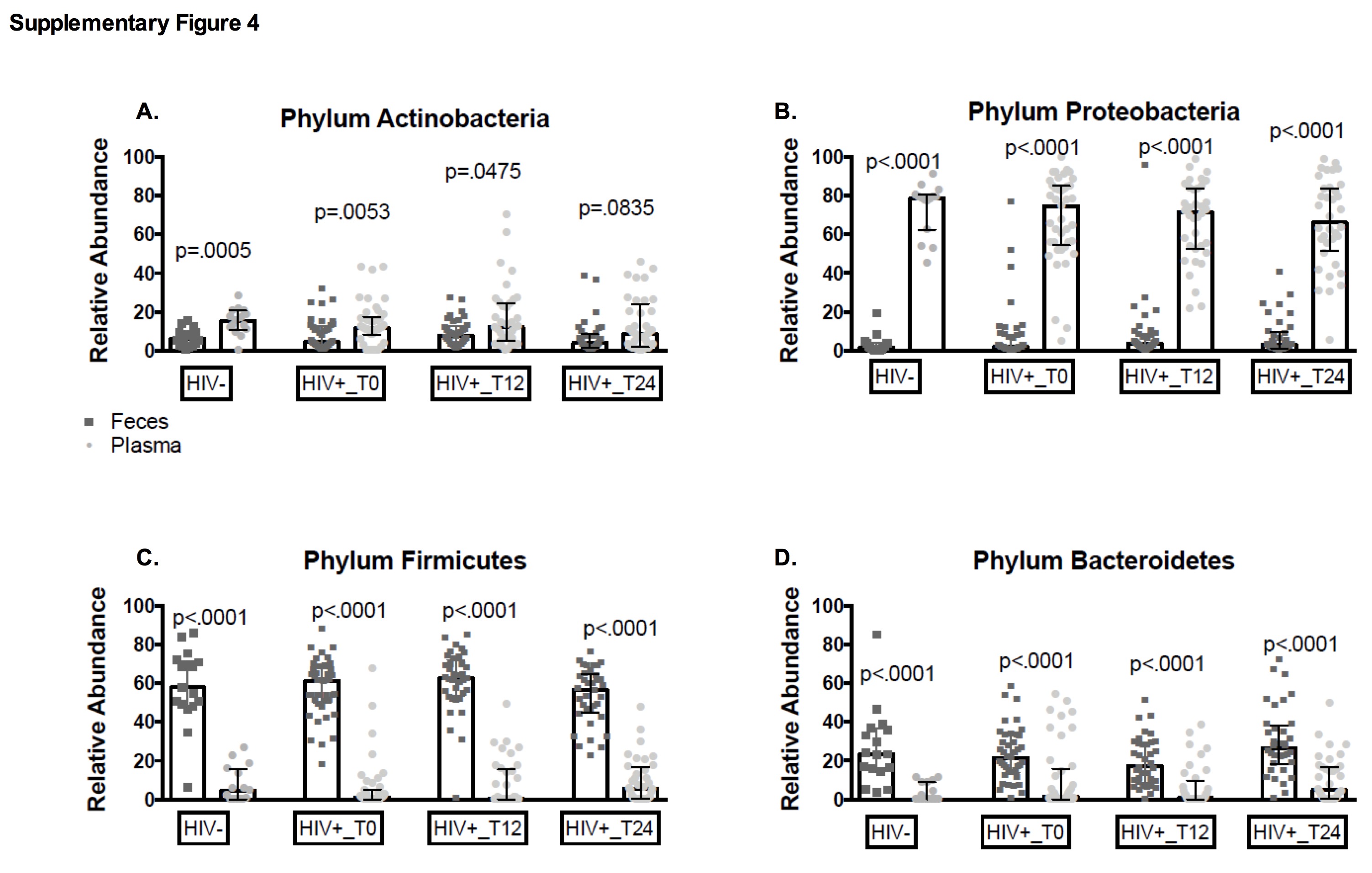

Supplement: Supplementary Figure 4 — Fecal and Plasma Phyla Distribution. The figure shows the relative abundance ratio between fecal (left bars) and plasma (right bars) samples among the most 4 representative phyla: Actinobacteria (A), Proteobacteria (B), Firmicutes (C), and Bacteroidetes (D) for each group: HIV negative controls, HIV+ T0 (baseline), HIV+ T12 (after 12 months the introduction of cART) and HIV+ T24 (after 24 months the introduction of cART). (A,B) The relative abundance of Actinobacteria and Proteobacteria phyla was higher in plasma (right bars) than in feces (left bars) in all the study groups. (C,D) The relative abundance of Firmicutes and Bacteroidetes phyla was lower in plasma (right bars), as compared to feces (left bars), in both healthy controls and HIV-infected patients prior and after cART introduction. P-values are not adjusted for multiple comparisons. [file Image_4.JPEG]
