# Supplementary material for: Long-Term Suppressive cART Is Not Sufficient to Restore Intestinal Permeability and Gut Microbiota Compositional Changes
Source: Front Immunol. 2021 Feb 26;12:639291. doi: 10.3389/fimmu.2021.639291 (PMC7952451; doi:10.3389/fimmu.2021.639291)
Supplement: Supplementary Table 1 — Viro-immunological parameters, T-cell phenotypes, microbial translocation, and gut barrier markers in 41 HIV-infected patients starting a first cART regimen. [file Table_1.DOCX]

**Supplementary Table 1. Viro-immunological parameters, T-cell phenotypes, microbial translocation and gut barrier markers in 41 HIV+ patients starting a first cART regimen**

|  | **T0** | **T12** | **T24** | **T0 vs T12** | **T0 vs T24** | **3-way comparison** |
| --- | --- | --- | --- | --- | --- | --- |
| ***Viro*-*immunological* *parameters*** |  |  |  |  |  |  |
| **CD4 T-cell count, cell/mmc (IQR)** | 342 (259-431) | 539 (442-686) | 618 (464-787) | **<0.0001** | **<0.0001** | **<0.0001** |
| **CD8 T-cell count, cell/mmc (IQR)** | 1000 (874-1272) | 860 (648-1124) | 786 (660-1069) | 0.050 | 0.071 | 0.322 |
| **CD4/CD8 ratio, (IQR)** | 0.31 (0.24-0.44) | 0.62 (0.48-0.78) | 0.73 (0.56-1.06) | **<0.0001** | **<0.0001** | **<0.0001** |
| **HIV-RNA, cp/ml, (IQR)** | 63734 (22751-205583) | <40 | <40 | **<0.0001** | **<0.0001** | **<0.0001** |
| **T-*cell* *phenotypes*** |  |  |  |  |  |  |
| **CD38+CD8+, % (IQR)** | 12 (6-18) | 2 (1-3) | 2 (1-3) | **<0.0001** | **<0.0001** | **<0.0001** |
| **CD38+CD45R0+CD8+, % (IQR)** | 5 (3-12.75) | 1 (0-1) | 1 (0.50-1) | **<0.0001** | **<0.0001** | **<0.0001** |
| **CD127+CD4+, % (IQR)** | 10 (8-12) | 17 (12.75-21) | 20 (16-25) | **<0.0001** | **<0.0001** | **<0.0001** |
| **CD127+CD8+, % (IQR)** | 25 (21-32) | 24 (19.75-28) | 26 (21-29.5) | 0.238 | 0.987 | 0.326 |
| **CD45R0+CD8+, % (IQR)** | 21 (16.75-31.75) | 13 (9-17.25) | 13 (9-16.5) | **<0.0001** | **0.0011** | **<0.0001** |
| **CD45RA+CD8+, % (IQR)** | 19 (13.25-23) | 19 (14.75-22) | 21 (16-25) | 0.569 | 0.820 | 0.549 |
| **CD45RA+CD4+, % (IQR)** | 6 (3-9) | 7.5 (5.75-14.25) | 12 (7-18.5) | **<0.0001** | **0.0012** | **0.0002** |
| ***Microbial translocation and gut barrier markers*** |  |  |  |  |  |  |
| **16S rDNA, cp/μL (IQR)** | 125 (56.5-237.5) | 90 (49-279) | 145 (54-311) | 0.546 | 0.891 | 0.721 |
| **sCD14, μg/mL (IQR)** | 5.13 (4.11-7.78) | 5.90 (49-3.89-9.67) | 5.58 (4.23-7.75) | 0.238 | 0.784 | 0.549 |
| **EndoCAb, MMU/mL (IQR)** | 45.8 (19.2-82.2) | 49 (21.4-75) | 90.2 (74.8-124.8) | 0.510 | **0.0004** | **0.0001** |
| **I-FABP, pg/mL (IQR)** | 664 (392-803) | 978 (595-1929) | 828 (534-1202) | **0.002** | 0.057 | **0.039** |
| **Fecal calprotectin, mcg/g (IQR)** | 95.7 (27.7-208) | 29.3 (16.4-141) | 47.7 (21.4-82.05) | **0.027** | 0.097 | 0.102 |
| **Lac/Man ratio, (IQR)** | 0.017 (0.012-0.052) | 0.032 (0.022-0.037) | n/a | 0.658 | n/a | 0.658° |

Note: *Data are median (IQR), statistical analysis Friedman Test, with Dunn’s multiple comparison; Wilcoxon matched pair test for comparison between T0 vs T12, and T0 vs T24; IQR: Interquartile range. 16s rDNA: 16s ribosomal DNA, sCD14: soluble CD14, EndoCAb: anti-endotoxin core antibodies, I-FABP: intestinal fatty acid binding protein. ° Wilcoxon signed rank test.
