# Supplementary material for: Long-Term Suppressive cART Is Not Sufficient to Restore Intestinal Permeability and Gut Microbiota Compositional Changes
Source: Front Immunol. 2021 Feb 26;12:639291. doi: 10.3389/fimmu.2021.639291 (PMC7952451; doi:10.3389/fimmu.2021.639291)
Supplement: Supplementary Table 2 — Viro-immunological parameters, T-cell phenotypes, microbial translocation/gut barrier markers in HIV-infected subjects treated with different classes of antiretrovirals. [file Table_2.DOCX]

|  | **T0** | | | | **T12** | | | | **T24** | | | |
| --- | --- | --- | --- | --- | --- | --- | --- | --- | --- | --- | --- | --- |
|  | **NNRTI** | **PI** | **INSTI** | **p** | **NNRTI** | **PI** | **INSTI** | **p** | **NNRTI** | **PI** | **INSTI** | **p** |
| ***Viro*-*immunological***  ***parameters*** |  |  |  |  |  |  |  |  |  |  |  |  |
| CD4 T-cell count  cell/mmc | 366  (291-438) | 268  (141-337 | 373  (316-633) | 0.05 | 562  (469-664) | 494  (388-65) | 643  (421-1406) | 0.4 | 566  (465-737) | 701 (505-966) | 638  (452-837) | 0.4 |
| CD8 T-cell count  cell/mmc | 1015  (892-1218) | 899  (563-1412) | 1001  (886-1347) | 0.7 | 959  (743-1141) | 670  (654-1252) | 886  (627-1396) | 0.5 | 786  (706-1198) | 851  (607-948) | 670  (580-840) | 0.5 |
| CD4/CD8 ratio | 0.3  (0.2-0.5) | 0.3  (0.3-0.33) | 0.43  (0.23-0.5) | 0.2 | 0.61  (0.5-0.8) | 0.7  (0.3-0.8) | 0.7  (0.6-1.4) | 0.6 | 0.70  (0.5-0.8) | 1.11  (0.6-1.5) | 1.06  (0.6-1.1) | 0.06 |
| HIV-RNA  cp/ml | 63783  (30965-195950) | 74000  (14347-451427) | 176256  (8592-674261) | 0.9 | <40 | <40 | <40 | na | <40 | <40 | <40 | na |
| ***T*-*cell* *phenotypes*** |  |  |  |  |  |  |  |  |  |  |  |  |
| CD38+CD8+, % | 12 (6-23) | 16 (15-18) | 6 (5-8) | 0.05 | 2 (1-4.5) | 2 (1-3) | 1.5 (1-2) | 0.36 | 3 (2-4) | 2 (1-2) | 2 (1-2) | **0.03** |
| CD38+CD45R0+CD8+, % | 5 (3-12.75) | 11 (8.75-13.25) | 3.5 (3-7.5) | 0.1 | 1 (0.50-1) | 1 (0-1.5) | 0 (0-0.75) | 0.16 | 1 (1-2) | 1 (0-1) | 1 (0-1) | 0.1 |
| CD127+CD4+, % | 10 (5.5-12) | 11 (8-12) | 18 (9-26) | 0.07 | 17 (13-21) | 17 (11-20) | 23 (16-33) | 0.3 | 19 (16-24) | 21 (15.5-27.5) | 24 (12-30) | 0.5 |
| CD127+CD8+, % | 25 (21-32) | 25 (22-30) | 28 (20-33) | 0.1 | 27 (22-29) | 23 (18-33) | 24 (15-24) | 0.5 | 28 (23-31) | 26 (17-28) | 21 (24-19) | **0.04** |
| CD45R0+CD8+, % | 21 (17-31) | 33 (14-39) | 21 (17-26) | 0.5 | 13 (10-14) | 15 (9-25) | 10 (5-15) | 0.3 | 14 (11-19) | 10 (6-13) | 9 (6-16) | 0.07 |
| CD45RA+CD8+, % | 21 (16.-24) | 15 (12-22) | 14 (9-20) | 0.050 | 20 (16-22) | 15 (10-21) | 17 (6-27) | 0.4 | 22 (18-25) | 20 (17-25) | 16 (14-25) | 0.5 |
| CD45RA+CD4+, % | 6 (3-8) | 3 (2-10) | 8 (4-14) | 0.3 | 7 (5-14) | 6 (4-15) | 15 (6-19) | 0.6 | 12 (7-19) | 13 (6-24) | 10 (7-13) | 0.8 |
| ***Microbial translocation***  ***and gut barrier markers*** |  |  |  |  |  |  |  |  |  |  |  |  |
| 16S rDNA  cp/μL | 126  (49-195) | 125  (49-264) | 202  (97-498) | 0.3 | 82  (49-297) | 138  (49-417) | 62  (49-230) | 0.6 | 144  (49-299) | 89  (59-310) | 109  (49-276) | 0.9 |
| sCD14  μg/mL | 5  (4-7) | 6  (4-9) | 7  (6-14) | 0.2 | 5 (4-8) | 5  (4-9) | 11  (7-12) | 0.09 | 6  (5-8) | 5  (4-9) | 6  (4-8) | 0.1 |
| EndoCAb  MMU/mL | 62  (18-86) | 45  (18-100) | 62  (33-88) | 0.9 | 41  (18-64) | 40  (18-95) | 56  (25-117) | 0.6 | 102  (81-121) | 83  (52-134) | 73  (55-102) | 0.2 |
| I-FABP  pg/mL | 800  (518-1365) | 761  (560-1048) | 595  (278-933) | 0.7 | 844  (402-1868) | 955  (677-1557) | 2544  (1013-3836) | 0.267 | 941  (625-1380) | 1012  (637-1562) | 752  (383-1757) | 0.8 |
| Fecal calprotectin  mcg/g | 90  (25-247) | 96  (19-177) | 49.6  (28-167) | 0.7 | 21  (16-89) | 62  (19-158) | 29  (24-157) | 0.359 | 45  (23-77) | 22  (5.-240) | 53  (33-96) | 0.6 |
| Lac/Man ratio | 0.02  (0.01-0.05) | 0.01  (0.002-0.06) | 0.02  (0.006-0.035) | 0.5 | 0.03  (0.02-0.04) | 0.03  (0.02-0.04) | 0.03  (0.03-0.04) | 0.5 | na | na | na | na |

**Supplementary Table 2: Viro-immunological parameters, T-cell phenotypes and microbial translocation/gut barrier markers in HIV-infected subjects treated with different classes of antiretrovirals**

Data analyzed by Kruskal-Wallis (one-Way Anova) to assess possible differences between groups treated with different cART class. Data presented as median, interquartile range. NNRTI: non-nucleoside reverse-transcriptase inhibitor; PI: protease inhibitor; INSTI: integrase strand transfer inhinitor. 16s rDNA: 16s ribosomal DNA, sCD14: soluble CD14, EndoCAb: anti-endotoxin core antibodies, I-FABP: intestinal fatty acid binding protein. Lac/Man: lactulose/mannitole ratio; NA: not applicable
